# CSF Biomarkers and Neuropsychological Profiles in Patients with Cerebral Small-Vessel Disease

**DOI:** 10.1371/journal.pone.0105000

**Published:** 2014-08-22

**Authors:** Peter Hermann, Carlos Romero, Christian Schmidt, Clemens Reis, Inga Zerr

**Affiliations:** 1 Department of Neurology, Dementia Research Group, Georg-August University Goettingen, Goettingen, Germany; 2 German Center for Neurodegenerative Diseases (DZNE), Bonn, Germany; 3 Department of Neuroradiology, and # Department of Neuropathology, FLENI, Buenos Aires, Argentina; Oregon Health & Science University, United States of America

## Abstract

Despite existing criteria, differential diagnosis of Vascular Dementia (VD) and Alzheimer's disease (AD) remains difficult. The aim of this study is to figure out cognitive and biomarker profiles that may help to distinguish between VD, AD and AD + Cerebral Small Vessel Disease (CSVD). We examined a cohort of patients with CSVD (n = 92). After stratification of cognitive impaired patients (n = 59) using the standard CSF beta-amyloid 42/40 ratio cut-off point of 0.975, we obtained two groups which differed with respect to several features: 32 patients with normal beta-amyloid 42/40 ratio (>0.975) showed markedly impaired blood-brain-barrier function as indicated by an elevated albumin ratio (median 8.35). They also differed in cognitive profiles when compared to 27 patients with AD typical beta-amyloid ratio and normal albumin ratio. We also enrolled an additional group of patients with AD (no significant CSVD on MRI, n = 27) which showed no impairment of the blood-brain-barrier. We showed a negative correlation between the albumin ratio and executive cognitive function (p = 0.016) and a negative correlation between memory function and typical AD markers like Tau (p = 0.004) and p181-Tau (p = 0.023) in our cohort. We suppose that the group of patients with normal beta-amyloid ratio represents VD while patients in the other groups represent AD+CSVD and pure AD. Our results support the idea that a dysfunction of the blood-brain-barrier might be contributing factor in the development of cognitive decline in CSVD as it seems to be of more importance than the severity of white matter lesions.

## Introduction

There is an ongoing discussion on pathophysiology, diagnostic criteria and phenotypic diversity of vascular dementia (VD). It is supposed to be the second leading cause of dementia after Alzheimer's disease (AD) but its prevalence remains unclear since studies differ from 0.03-58% in autopsy series[1]. Several criteria have been developed to diagnose VD in a clinical setting and for research studies. Criteria like NINDS-AIREN[2] refer to different entities of VD including the use of imaging (territorial or lacunar strokes), clinical features and causal relation with cerebral ischemic stroke. Dementia following stroke is easy to define, but the diagnosis of subcortical ischemic dementia caused by small vessel disease remains controversial. Over the last years many studies have reported that vascular risk factors contribute to both, VD and AD. Many cases of dementia are considered mixed VD and AD and vascular risk factors might play a major role [3]. Current clinical criteria allow a huge overlap between the aforementioned entities [4] and a distinction as separate entities is not always clearly made [5]. Mixed forms of AD and VD, especially dementia caused by CVSD, do not appear in most criteria and therefore they are not considered in most studies [6].

Subcortical white matter lesions (WML) have been reported to cause VD [7]. The severity of WML on MRI can be measured through qualitative visual rating scales like the Age Relate White Matter Changes (ARWMC) scale [8]. Neuropsychological testing is supposed to classify CSVD and AD+CSVD (mixed dementia) correctly [9]. Strong impairment of executive cognitive function with working memory less affected seems to indicate subcortical dementia. Specific neuropsychological batteries are needed for diagnosis and for research to make studies comparable [10].

### CSF Biomarkers

CSF biomarkers are becoming more important in the differential diagnosis of dementia and degenerative neurological diseases. Tau isoforms and beta-amyloid peptides are in focus and many studies attempt to define an AD-specific profile [11]. A ratio of phosphorylated tau to total is supposed to allow AD diagnosis with high specificity against other dementia [12]. A huge amount of studies also demonstrates that the ab 42/40 ratio (ab-ratio) clearly differentiates patients with AD from patients with other dementia. An ab-ratio cut-off point of 0.975 has been calculated to correctly classify AD versus patients with other cause for dementia with a specificity of 90% [13]. Ab peptides and ratios are also discussed to indicate participation of AD-pathology in patients with clinical possible or probable VD [14]. However, a CSF biomarker with high specificity for VD in differential diagnosis of dementia has not been established yet. Some studies have shown that the neurofilament light protein in CSF is related with volume of WML and is probably a marker for axonal damage due to small vessel disease [15], [16]. While correlation between CSF biomarkers and disease progression in AD has been shown, for patients with CVSD it still needs to be examined.

### Objectives of this study

The main objective of this study is to identify parameters such as neuropsychological test profiles and/or CSF biomarkers which will allow the discrimination between patients with sole CSVD from those with AD+CSVD and pure AD. Another objective was to identify other than neuropsychologic diagnostic markers, which differentiate VD from CSVD without dementia.

## Methods

### Study design and patients

For this case control study, inclusion criteria was the presence of white matter lesion (score on ARWMC-Scale > = 3). Patients were referred to the Department of Neurology, Georg-August University, Goettingen, Germany and received MRI for various diagnostic reasons, e.g. gait disturbance, headaches, vertigo or cognitive deficits. However, dementia was not necessarily the primary reason for the MRI, which was performed in clinical routine. Patients suffering from major depression, inflammatory disease of the central nervous system, metabolic encephalopathy, recent stroke (less than 8 weeks), territorial infarction, space occupying lesion or cerebral hemorrhage were excluded. Further, only patients who received lumbal puncture on diagnostic purpose (exclusion of inflammatory or neoplastic disease) were included in this study.

Ninety-two patients, 44 males and 48 females, aged 37 to 88 years were included. All patients were examined by a study physician (P.H., C.R.), blind to results of CSF analysis. Medical history was collected. All human studies were approved by the local ethics committee in Goettingen (Ethic committee at the Medical University of Goettingen, Germany (No. 34/9/07)). All participants or respectively their legal representatives signed informed consent to participate in this study.

### Imaging, neuropsychological assessment and CSF Biomarkers

All patients received T2 or FLAIR weighted 3 Tesla MRI using standard protocols for dementia diagnosis or, if suspected, for ischemic cerebral disease including diffusion weighted images. An experienced neuroradiologist (C.R.), rated the scans using the ARWMC-Scale and analyzed them for pathological findings matching exclusion criteria. Patients received a neuropsychological assessment including the MMSE and a German version of the Cambridge Cognitive Examination battery (CAMCOG) from the CAMDEX-R [17]. For further analysis we focused on two subscales for memory and executive function. CSF analyses for tau protein and amyloid ratio [(abeta1-42/abeta1-40)*10)] have been performed using standard methods in the research laboratory of the dementia research group, University of Goettingen. Analysis of standard CSF parameters and albumin ratio [Qalb  =  (CSF albumin/serum albumin)*10^3^] have been performed by the Neurochemistry Laboratory at the University Hospital according to standard methodology.

### CAMCOG ratio

We looked for a score that would allow us to compare performances in executive function and memory function between groups of heterogenous global cognitive condition. Dividing subscale sores from CAMCOG memory function by CAMCOG executive function results in a ratio [mem/exec]. This relative value is independent from absolute scores and was used as a mathematical tool in our group comparisons. Our aim was not to establish a new cognitive score and therefore no validation has been performed. A higher CAMCOG ratio indicates better performance in memory function and worse performance in executive function towards a lower CAMCOG ratio.

### Groups and controls

To separate those individuals from our cohort that did not show relevant cognitive decline, we used the Mini Mental Status Examination (MMSE). Freely accessible population based reference data from the ETH, Zurich were used as reference. All patients with MMSE scores not lower than 1.5 standard deviations below reference (adjusted for age, sex and education) were considered cognitively unimpaired have been marked as (‘healthy’) controls (n = 33).

**Figure 1:**
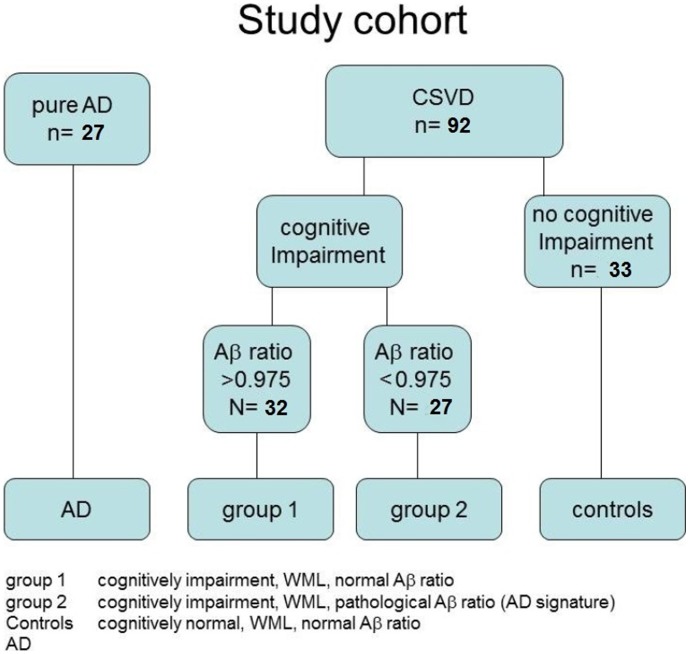
Stratification of study cohort.

Due to overlap, patients with AD+CSVD regularly fulfill criteria for AD and for VD. So we analyzed the data of 59 cognitively impaired individuals from our cohort after stratification into two groups using the CSF ab-amyloid-ratio and a cut-off point at 0.975 [13]. Individuals with normal CSF ab-ratio were assigned as group1 (none AD CSF signature, n = 32) and individuals showing a decreased ab-ratio as group2 (AD CSF signature, n = 27). For comparative CSF analyses including cases of ‘pure AD’, we selected an additional group of patients with clinical diagnosis of AD showing no relevant WML on MRI (ARWMC < 3). Those patients were recruited in another prospective study on risk factors for disease progression in AD (n =  27). An overview on our groups is given in figure 1.

### Statistical analysis

All statistical analysis have been performed using SSPS 19.0 software. Comparisons between multiple groups were done using ANOVA respectively ANCOVA adjusted for age and gender and for Post hoc analysis Turkey-HSD. Correlations between two variables were calculated with the Spearman Rank Order Correlation. P was considered significant when < 0.05.

## Results

The median age at enrollment in patients with CSVD was 73 years and did not differ significantly from those with AD no CSVD (72). Within CSVD, controls were younger than group1 (p = 0.03) and group2 (p<0.001). The median ARWMC score was 9.0 points (range 3–30). Group1 had a non-significant higher median score on the ARWMC scale than controls and group2. The median CAMCOG ratio had a value of 1.15. There are less female patients in the control group (33.4%) and group1 (40.6%) than in group2 (66.7%) and AD group (66.7%). The data is shown on table 1.

**Table 1:** Description of groups.

	Gender		Age [years]		ARWMC [score]		MMSE [score]	
	female	male	Median	min–max	Median	min–max	Median	min–max
**Controls** (n = 33)	13	20	69.0	37–83	8.0	3–19	29.0	27–30
**Group 1** (n = 32)	13	19	74.0	46–88	10.0	3–30	23.5	9–27
**Group 2** (n = 27)	18	9	78.0	65–87	9.0	3–16	23.0	11–26
**AD no CSVD** (n = 27)	18	9	72.0	53–88			22.0	11–27

**Figure 2:**
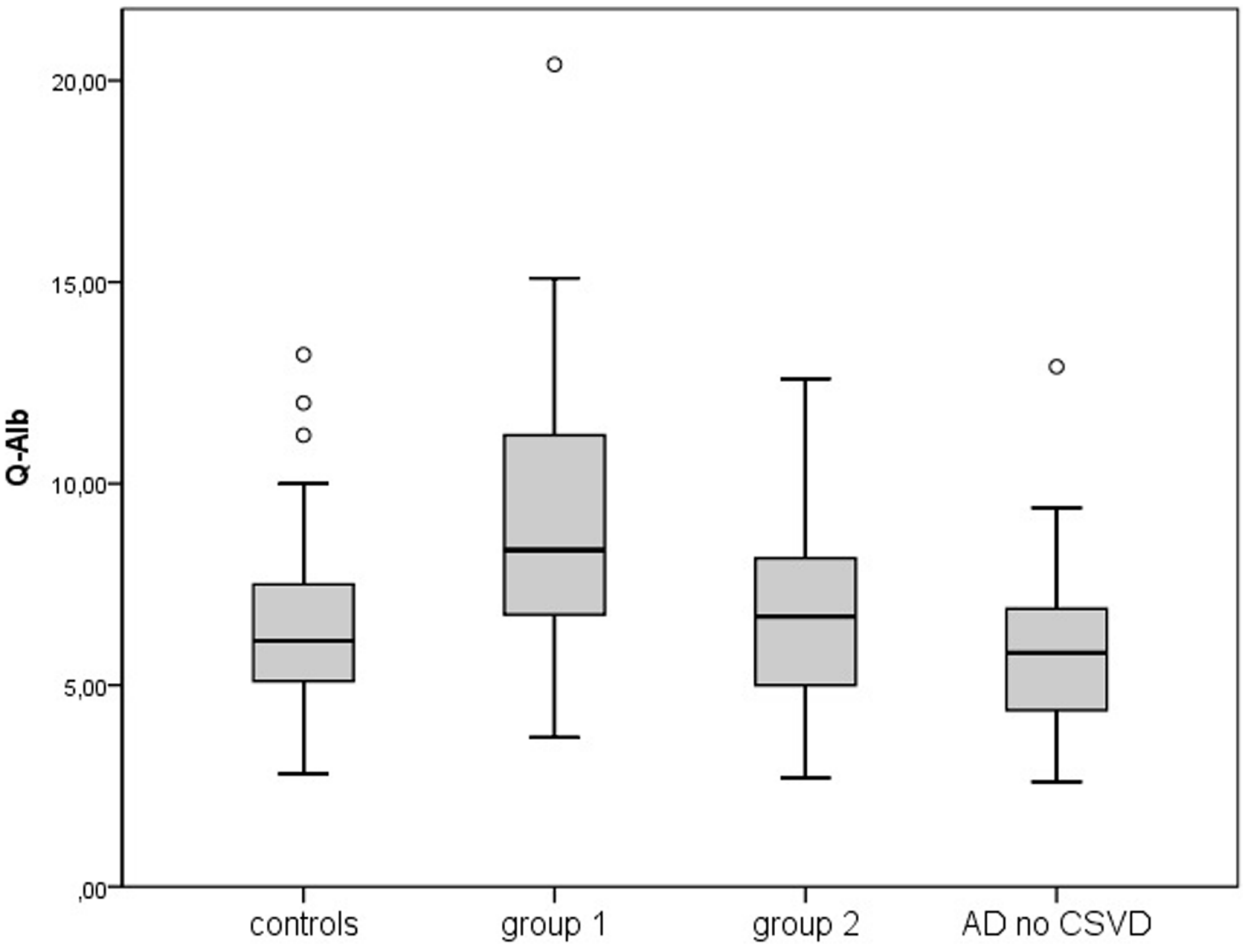
CAMCOG [subscores] in groups.

Concerning CSF parameters, the median albumin ratio in our cohort was 6.4, which is age-corrected a normal level (age group 70-80). The most striking result was the difference of the albumin ratio between groups. It was significantly elevated in group1 (8.35) when compared to group2 (p = 0.013), controls (p = 0.002) and AD no CSVD (p<0.001) as shown in figure 2.

Levels of tau protein and p-tau protein in CSF were elevated in group2 as well as AD no CSVD with high significance when compared to group 1 and controls in a similar way for both proteins (p<0.001). We saw no significant difference between controls and group1. Group2 also showed a lower median tau level than AD no CSVD but when ANOVA has been performed, no significant difference between mean levels could be observed. The data are shown on table 2. The results from ANOVA are summarized on table S1.

**Table 2:** Description of CSF biomarker and CAMCOG in groups.

	Tau [pg/ml]		P181-Tau [pg/ml]		Alb. ratio [csf/serum]	
	Median	min–max	Median	min–max	Median	min–max
**Controls**	156	<75 – 635	39	16 – 78	6.10	2.8 – 13.2
**Group 1**	148	<75 – 1033	34	16 – 87	8.35	3.7 – 28.6
**Group 2**	390	<75 – 2120	94	15 – 224	6.70	2.7 – 12.6
**AD no CSVD**	610	195 – 2022	101	52 – 295	5.8	2.6 – 12.9

	Mem. function [score]		Exec. function [score]		Ratio [mem/exec]	
	Median	(min–max)	Median	(min–max)	Median	(min–max)
**Controls**	21.0	14 – 25	19.5	9 – 27	1.10	0.63 – 2.44
**Group 1**	17.9	5 – 25	11.0	6 – 19	1.30	0.75 – 2.67
**Group 2**	11.5	4 – 21	11.5	4 – 23	1.0	0.31 – 2.33

Due to our stratification criteria, group1 and group2 showed lower scores on all neuropsychiatric scales than controls, no significant difference between group1, group2 has been found regarding MMSE scores and scores of CAMCOG executive functions. CAMCOG memory function scores in group2 were lower in group1 (p = 0.003). The CAMCOG ratio was higher in group1 when compared to group2 (p = 0.013) and controls (p = 0.037). The data are shown on table 2. The results from ANOVA are summarized on table S1.

In another step, we analyzed CSF biomarker correlations in our cohort.

Higher scores on the ARWMC scale were associated with lower score in the MMSE (p = 0.043) and CAMCOG executive function (p = 0.082) and higher albumin ratio (p = 0.051). These associations showed no or at highest borderline significance. CSF tau and p-tau levels were strongly correlated (p<0.001). Higher tau levels were also associated significantly with higher age (p = 0.003) as well as lower MMSE scores (p<0.001) and memory function (p = 0.004). We saw no significant correlation between tau and executive function or CAMCOG ratio. The albumin ratio showed negative correlation with CAMCOG executive function (p = 0.016) while it was not associated significantly with age, tau and memory function. The results are shown on table S2. Biomarker correlations were also calculated in each group. All results showed tendencies similar to results from the cohort but were less or non-significant (data not shown).

## Discussion

In this study, we analyzed a cohort of patients with CVSD with respect to clinical data, neuroimaging, CSF biomarkers and neuropsychological test profiles. Among these, we identified a great variety of cognitive disturbances, which ranged from cognitively normal individuals to severely impaired dementia patients. With respect to CSF biomarker profiles, we identified a cohort of patients who displayed an „AD-signature“, which is defined by low ab-ratio and high levels of protein tau and its phosphorylated isoforms (group2). In neuropsychological tests, they had greater impairment of memory functions than executive functions. Taking all these results into account, we have to discuss that these patients most likely represent a subgroup which is very close to pure AD patients. Still, the significance of WML in MRI in these patients is not clarified to date, but seems to be of minor importance or could be a by chance finding.

The characteristics of the other group1 are summarized as following: slightly higher WML score, normal tau and p-tau levels in CSF but disturbed blood-brain-barrier as determined by the albumin ratio, severely impaired executive cognitive functions. We have to discuss, that most likely the mechanisms of pathogenesis that are involved here are different from those in group2. This idea is supported by the fact that in our cohort the albumin ratio (elevated in group1) is associated with executive function while tau and ptau (elevated in group2) are associated with memory function. Patients in group1 are more likely to represent patients with pure CSVD or VD.

In recent years, there has been a considerable amount of studies on biomarker identification and validation in patients with AD, VD and AD plus WML. Only few studies with small cohorts are available on the albumin ratio in this context, but they also revealed that in VD the ratio is elevated and the blood-brain-barrier is thus impaired [18]. Other studies which analyze multiple parameters (albumin ratio and common CSF biomarkers for dementia) are not available. Our CSF results are in accordance with the current ideas on pathogenesis of small-vessel disease of the brain: small vessel structural changes are characterized by vessel wall thickening leading to a structural breakdown and perivascular changes like edema and enlarged perivascular spaces [19]. The blood-brain-barrier as an endothelial function is affected early and its breakdown is a leading factor in progression of the pathological process. Elevated albumin and other serum proteins have been found in CSF and in the brain of patients with vascular dementia [18].

Controversial data are obtained with regard to the significance of WML in MRI and clinical syndrome. Some support the idea, that severity or progression of WML in an elderly population is an independent predictor for the occurrence of cognitive decline [20]–[22]. Other studies showed that the presence of WML in cognitively impaired patients had no effect on the progression rate of dementia [23]. Of interest and with respect to the brain imaging data, we hypothesize that WML represent abnormalities, which do not necessarily indicate development of cognitive decline. In patients from group1, WML might correspond to microvascular pathology and may lead to dementia, whereas in patients from group2, we must assume another pathogenesis of cognitive decline. The role of WML and their relevance as a marker for pure vascular dementia also remains uncertain, since the control group represents a cohort of patients with WML showing no cognitive decline. These conclusions are substantiated by our findings on neuropsychological tests. We only found a tendency of borderline significance between severity of WML and cognitive performance. In addition, the difference of the severity of WML between cognitively healthy and impaired patients did not turn out to be significant.

With regard to the literature, we assume that cognitive decline and WML are associated and may share pathogenic pathways, but WML do not seem to be the best marker to differentiate VD from other dementia. Therefore we have to search for other parameters that are linked to the pathophysiology of cerebral small vessel disease. Markers of blood brain barrier function like the albumin ratio seem to be promising targets for prospective studies.

**Figure 3:**
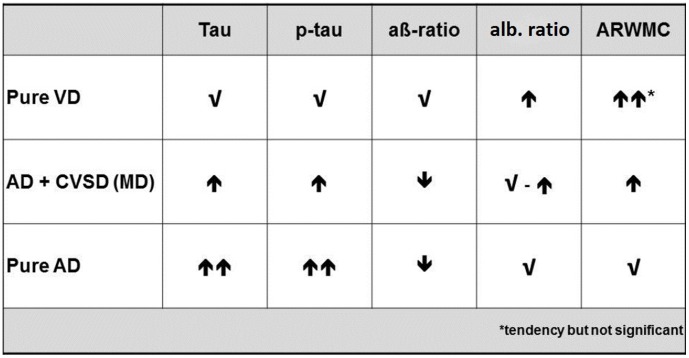
Albumin ratio [CSF/serum] in groups.

In summary, we found significant differences between CSF biomarker levels and neuropsychological test profiles in two groups of cognitive impaired and demented patients, both showing white matter lesions on MRI. We assume that these groups represent patients with pure VD and AD+CSVD. Studies on therapy and diagnostic markers in AD and VD still suffer from a very difficult differentiation between these two entities and the common case of a mixed disease. In recent years, CSF biomarker proteins have become a widely used diagnostic instrument. Our results demonstrate the importance of these markers. A combination of neuropsychological profile, albumin ratio and common CSF biomarkers for Alzheimer's disease could help to distinguish between pure dementia due to cerebral small vessels disease, Alzheimer's disease and AD + CSVD (for a synopsis, see figure 3, patients from group1 are indicated as ‘pure VD’ and patients from group2 as ‘AD+CSVD’).

## Supporting Information

Table S1 **Multiple comparisons (ANOVA – adjusted for age and gender).**
(DOC)

Table S2 **Correlations in cohort CSVD (n  =  92).**
(DOC)
