# Supplementary material for: CSF Biomarkers and Neuropsychological Profiles in Patients with Cerebral Small-Vessel Disease
Source: PLoS One. 2014 Aug 22;9(8):e105000. doi: 10.1371/journal.pone.0105000 (PMC4141759; doi:10.1371/journal.pone.0105000)
Supplement: Table S1 — Multiple comparisons (ANOVA – adjusted for age and gender). (DOC) [file pone.0105000.s001.doc]

# Supporting Information Legends

**Table S1. Multiple comparisons (ANOVA – adjusted for age and gender)**

|  | **ANOVA (mean difference)** | |
| --- | --- | --- |
| **Age** | controls are younger than group 1: p = 0.030 (-7.02)  controls are younger than group 2: p < 0.001 (-11.07)  no significant differences between other groups | Kruskal–Wallis  one-way analysis of variance:  p = 0.002  → reject null hypothesis |
| **ARWMC** | no significant differences between controls, group 1 and group 2 | |
| **Albumin Ratio** | group 1 has a higher ration than controls: p = 0.002 (3.03)  group 1 has a higher ration than group 2: p = 0.013 (2.62)  group 1 has a higher ration than group 3: p < 0.001 (3.85)  no significant differences between other groups | Kruskal–Wallis  one-way analysis of variance:  p < 0.001  → reject null hypothesis |
| **CSF Tau** | controls have a lower level than group 2: p < 0.001 (-382.73)  controls have a lower level than AD no CSVD: p < 0.001 (-456.66)  group 1 has a lower level than group 2: p < 0.001 (-337.66)  group 1 has a lower level than AD no CSVD: p < 0.001 (-411.59)  no significant difference between group 2 and AD no CSVD  no significant difference between controls and group 1 | Kruskal–Wallis  one-way analysis of variance:  p < 0.001  → reject null hypothesis |
| **CSF P181 Tau** | controls have a lower level than group 2: p < 0.001 (-54.47)  controls have a lower level than AD no CSVD: p < 0.001 (-70.46)  group 1 has a lower level than group 2: p < 0.001 (-56.23)  group 1 has a lower level than AD no CSVD: p < 0.001 (-72.32)  no significant difference between group 2 and AD no CSVD  no significant difference between controls and group 1 | Kruskal–Wallis  one-way analysis of variance:  p < 0.001  → reject null hypothesis |
| **CAMCOG mem** | group 1 has a higher score than group 2: p = 0.003 (4.13)  controls have a higher score than group 1 and 2 (p < 0.001)  for AD no CSVD no data has been obtained | Kruskal–Wallis  one-way analysis of variance:  p < 0.001  → reject null hypothesis |
| **CAMCOG exec** | controls have a higher score than group 1 and 2 (p < 0.001)  no significant difference between group 1 and 2  for AD no CSVD no data has been obtained | Kruskal–Wallis  one-way analysis of variance:  p < 0.001  → reject null hypothesis |
| **CAMCOG mem/exec** | group 1 has a higher ratio than controls: p = 0.037 (0.284)  group 1 has a higher ratio than group 2: p = 0.013 (3.44)  no significant difference between controls and group 2  for AD no CSVD no data has been obtained | Kruskal–Wallis  one-way analysis of variance:  p = 0.005  → reject null hypothesis |
