# Supplementary material for: CSF Biomarkers and Neuropsychological Profiles in Patients with Cerebral Small-Vessel Disease
Source: PLoS One. 2014 Aug 22;9(8):e105000. doi: 10.1371/journal.pone.0105000 (PMC4141759; doi:10.1371/journal.pone.0105000)
Supplement: Table S2 — Correlations in cohort CSVD (n = 92). (DOC) [file pone.0105000.s002.doc]

# Supporting Information Legends

**Table S2. Correlations in cohort CSVD (n = 92)**

| Variable 1 | Variable 2 | | Corr | P-value |
| --- | --- | --- | --- | --- |
|  |  | |  |  |
| **ARWMC** | Age | | 0.075 | 0.475 |
|  | MMSE | | -0.211 | 0.043* |
|  | CamCog exec | | -0.187 | 0.082 |
|  | CamCog mem | | -0.041 | 0.703 |
|  | CamCog ratio (mem/exec) | | 0.063 | 0.563 |
|  | Total – Tau | | -0.016 | 0.880 |
|  | P181 – Tau | | -0.050 | 0.651 |
|  | Albumin ratio | | 0.204 | 0.051 |
|  |  | |  |  |
|  |  | |  |  |
| **Albumin ratio [CSF/serum]** | Age | | 0.169 | 0.067 |
|  | MMSE | | -0,038 | 0.684 |
|  | CamCog exec | | -0.259 | 0.016** |
|  | CamCog mem | | -0.056 | 0.609 |
|  | CamCogratio (mem/exec) | | 0.192 | 0.156 |
|  | Total – Tau | | -0.034 | 0.715 |
|  | P181 – Tau | | -0.117 | 0.224 |
|  |  | |  |  |
| **Tau** |  | |  |  |
|  | Age | | 0.266 | 0.003** |
|  | MMSE | | 0.355 | < 0.001** |
|  | CamCog exec | | -0.132 | 0.246 |
|  | CamCog mem | | -0.308 | 0.004** |
|  | CamCogratio (mem/exec) | | -0.151 | 0.162 |
|  | P181 – Tau | | 0.805 | < 0.001** |
|  |  |  |  |  |
| Spearman Rank Order Correlation | | *p < 0.05 **p < 0.02 | | |
